# Vegetation and fire in lowland dry forest at Wa’ahila Ridge on O’ahu, Hawai’i

**DOI:** 10.3897/phytokeys.68.7130

**Published:** 2016-08-05

**Authors:** Pei-Luen Lu, John K. DeLay

**Affiliations:** 1 Department of BioResources, Da-Yeh University No.168, University Rd., Dacun, Changhua 51591, Taiwan; 2 Department of Botany, University of Hawai’i at Mānoa, 3190 Maile Way, Room 101, Honolulu, HI 96822, USA; 3 Honolulu Community College, University of Hawai’i 874 Dillingham Blvd., Honolulu Hawai’i 96817, USA

**Keywords:** Fire ecology, island ecology, O’ahu, restoration, vegetation ecology

## Abstract

Long-term ecological studies are critical for providing key insights in ecology, environmental change, natural resource management and biodiversity conservation. However, island fire ecology is poorly understood. No previous studies are available that analyze vegetative changes in burned and unburned dry forest remnants on Wa’ahila Ridge, Hawai’i. This study investigates vegetation succession from 2008 to 2015, following a fire in 2007 which caused significant differences in species richness, plant density, and the frequency of woody, herb, grass, and lichens between burned and unburned sites. These findings infer that introduced plants have better competitive ability to occupy open canopy lands than native plants after fire. This study also illustrates the essential management need to prevent alien plant invasion, and to restore the native vegetation in lowland areas of the Hawaiian Islands by removing invasive species out-planting native plants after fire.

## Introduction

Fire has a significant influence on global ecosystems (Pyne et al. 1996, Robertson et al. 2015). Fire influences global vegetation patterns, shapes species characteristics, and reduces the plant biomass (Riano et al. 2002, Bond et al. 2005). Some plants have developed traits to cope with recurrent fires as fire intolerant species (Pausas et al. 2004, Pausas and Keeley 2009). On the other hand, many plants belong to fire tolerant or fire resistant species such as Pondersosa Pine and Mountain Grey Gum tree (Knox and Clark 2005, Fitzgerald 2005, Kolb et al. 2007, Wesolowski et al. 2014). Understanding the factors that govern the distribution of tropical dry forest and invasive species has important implications for projecting the response of Hawai’i lowland landscapes to disturbance regimes and managing lowland dry forest ecosystems after fire.

Islands are good locations to study the influence of biological mechanisms on ecosystem-level properties (Carlquist 1974, Vitousek et al. 1995). Island ecosystems are susceptible to species invasion (Vitousek 1988, Glen et al. 2013, Meng et al. 2014). The Hawaiian Islands are the most remote archipelago on Earth and a hotspot for biodiversity (Wagner et al. 1990, 2012; Gustafson 2014). Although many lineages of plants are species rich in Hawai’i, few of them have been studied in detail (Mueller-Dombois and Fosberg 1998, Ziegler 2002, Mueller-Dombois and Boehmer 2013, Sherwood et al. 2014). One approach to the study of vegetation structure is to analyze diversity after a disturbance, such as a fire or a storm (Keeley 1986, Chen et al. 2013, Huston 2014, Pausas 2015, Burkle et al. 2015).

The mesic and dry forests of the Hawaiian Islands have been reduced due to habitat loss, including development, and the introduction and spread of invasive plants and animals (Gagne and Cuddihy 1999, Mayer et al. 2004, Wichman and Clark 2013). The island of O’ahu has the highest human population density of all the islands. One major concern is balancing development and natural resource conservation (State of Hawai’i data book 2007, 2015). Governmental and non-government organizations (NGOs) have been involved in preserving native ecosystems, the current vegetation structure on O’ahu, particularly in lowlands, is not well-preserved and thus very difficult to understand their structure. The 2007 fire on Wa’ahila Ridge provided an opportunity to study vegetation succession.

While agriculture and alien plant invasion are responsible for significant landscape transformations in the Hawai’i, fires cause dramatic and immediate changes to the original vegetation (Ziegler 2002). Wa’ahila Ridge, the southern side of Mānoa Valley, is characterized by a wetter winter and drier summer. It is a State Recreation Area on O’ahu. Little previous research on the vegetation ecology of this area has been reported. In 2007, there was a wild fire disturbance on Wa’ahila Ridge (State of Hawai’i 2007). This provided an opportunity to observe the post-fire vegetation structure and document the original vegetation on the unburned area. This research is a pioneer study to understand the effect of fire disturbance on the mesic forest on the lowland of O’ahu. This study was conducted after two fires occurred in the same location in the spring of 2015 (State of Hawai’i 2015).

In this study, the impacts of fire were examined by comparing the difference between burned and unburned plots. Plot level studies can provide an understanding of general post-fire sequences in forest ecosystems, landscape-scale monitoring, and analysis of post-fire recovery trajectories (Keeley and Keeley 1981, Keeley et al. 1981). Three hypotheses were tested. The first hypothesis is that the dominant species on unburned sites are woody plants because woody plants are the major component of in mesic forests in the lowlands; grass plants are dominant on burned sites because grasses are good pioneer species. The second hypothesis is that native plants are less frequent at burned sites because alien plants have better competitive ability to occupy open spaces after a fire even though the reproductive periods of native and alien plants are similar. The third hypothesis is that species richness and plant density are greater on unburned sites than burned sites because the recovery time has only been one year and thus not enough for some plants to recolonize the area.

## Methods

The study site is in Wa’ahila Ridge State Recreation Area on O’ahu in Hawai’i, at 21°18'1"N and 157°48'41"W. The average elevation of burned and unburned sites is about 100 m. Mean annual precipitation is 1039-2400 mm, with 64-80% falling October through March (Giambelluca et al. 2013). Mean annual temperature is 24°C. The soil type is an Andisol (Woodcock et al. 1999).

The date of fire was July 5, 2007. It burned for at least 6 hours and covered about 20 hectares (State of Hawai’i 2007). Seven years of data were collected on the following occasions: Oct. 2, 2008; Oct. 12, 2009; Nov. 1, 2010; Oct. 1, 2011; Oct. 7, 2012; Oct. 5, 2013; and Oct. 4, 2014. Fifty unburned and 50 burned 1 m^2^ plots were examined. The plots were randomly distributed within unburned and burned sites, but at the same site from year to year. Species richness, plant density, species dominance (based on frequency), and species diversity were examined on unburned area and burned areas.

Sampling followed the protocol of The Nature Conservancy of Hawai’i for long-term vegetation monitoring (The Nature Conservancy 1993). The number of individuals of each species expressed as percentage of total was used as a measure of relative density in the plots. Species composition and abundance was measured in each plot. Plant species richness, plant density, plant dominance, frequency of native plants, woody plants, forb plants, grass plants, and lichens were calculated for unburned and burned sites. Species composition was identified based on taxonomy and then documented. The only previous record of a major disturbance on Wa’ahila Ridge was a fire in 1888 (Hillebrand 1888); however, it is not clear whether the current study sites were involved. Means, standard deviations, and paired T-tests were used to compare burned and unburned sites, and were calculated with MiniTab 17.

## Results

One year after the fire, the proportion of alien species on unburned and burned sites was similar (Table 1). The dominant species on unburned sites is the alien woody plant, *Leucaena leucocephala*, and on burned sites, the alien invasive grass, *Urochloa maxima* dominates (Table 2). The species frequency indicates *Leucaena leucocephala* and lichens are high on unburned sites and *Urochloa maxima* is highest on burned sites (Table 3). The only woody species found in burned site is white lead-tree (*Leucaena leucocephala*). The frequency of this species at burned sites was much lower compared to unburned sites and partially indicates this species’ fire resistance. The only native grass, *Heteropogon contortus*, completely disappeared after this fire.

**Table 1.** Species re-sprouting and seed regenerating on unburned and burned sites one year after the fire. *Heteropogon contortus* and *Waltheria indica* are native species in Hawai’i. *Heteropogon contortus*, *Chloris barbata*, and *Urochloa maxima* are grass species. T is tree. G is grass. F is forb.

Unburned site	Type	Burned site	Type
*Haematoxylum campechianum*	T	*Stapelia gigantea*	F
*Leucaena leucocephala*	T	*Urochloa maxima*	G
*Chloris barbata*	G	*Leucaena leucocephala*	T
*Grevillea robusta*	T	*Chamaecrista nictitans*	F
*Heteropogon contortus*	G	*Agave attenuata*	F
*Pimenta dioica*	T	*Chloris barbata*	G
*Urochloa maxima*	G	*Waltheria indica*	F
*Lichens* (additional information)		*Fucraea foetida*	F
		*Kalanchoe pinnata*	F
		*Hyptis pectinata*	F
		*Senna septemtrionalis*	F
		*Murraya paniculata*	F

**Table 2.** Dominant species on burned and unburned sites one year after the fire.

Unburned site	Burned site
Woody plant: *Leucaena leucocephala*	Grass plant: *Urochloa maxima*
Individuals: 427	Individuals: 333
48/50 plots	49/50 plots

**Table 3.** Percentage (%) Frequency of species on unburned and burned sites one year after the fire.

Unburned site %		Burned Site %	
*Haematoxylum campechianum*	12	*Stapelia gigantea*	6
*Leucaena leucocephala*	96	*Urochloa maxima*	98
*Chloris barbata*	2	*Leuceana leucocephala*	10
*Grevillea robusta*	18	*Chamaecrista nictitans*	4
*Heteropogon contortus*	52	*Agave attenuata*	4
*Pimenta dioica*	4	*Chloris barbata*	2
*Urochloa maxima*	40	*Waltheria indica*	26
*Lichens* (additional information)	94	*Fucraea foetida*	22
		*Kalanchoe pinnata*	6
		*Hyptis pectinata*	2
		*Senna septemtrionalis*	2
		*Murraya paniculata*	8

**Figure 1.**
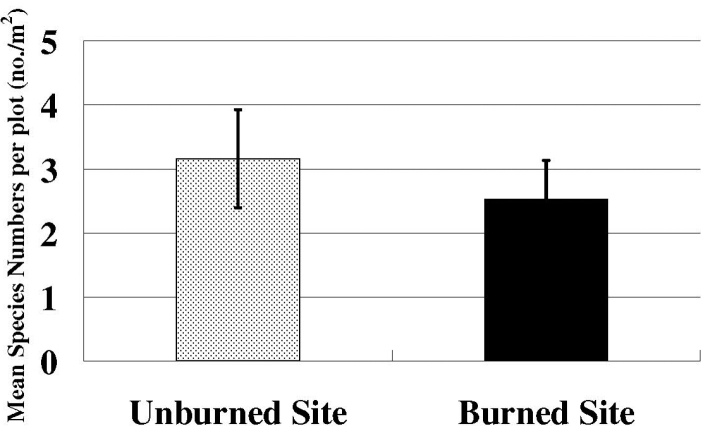
The species richness on the unburned site and the burned site per 1 m^2^. Error bars are ± SD (n=50). T-value= 4.70, P-vale < 0.001.

**Figure 2.**
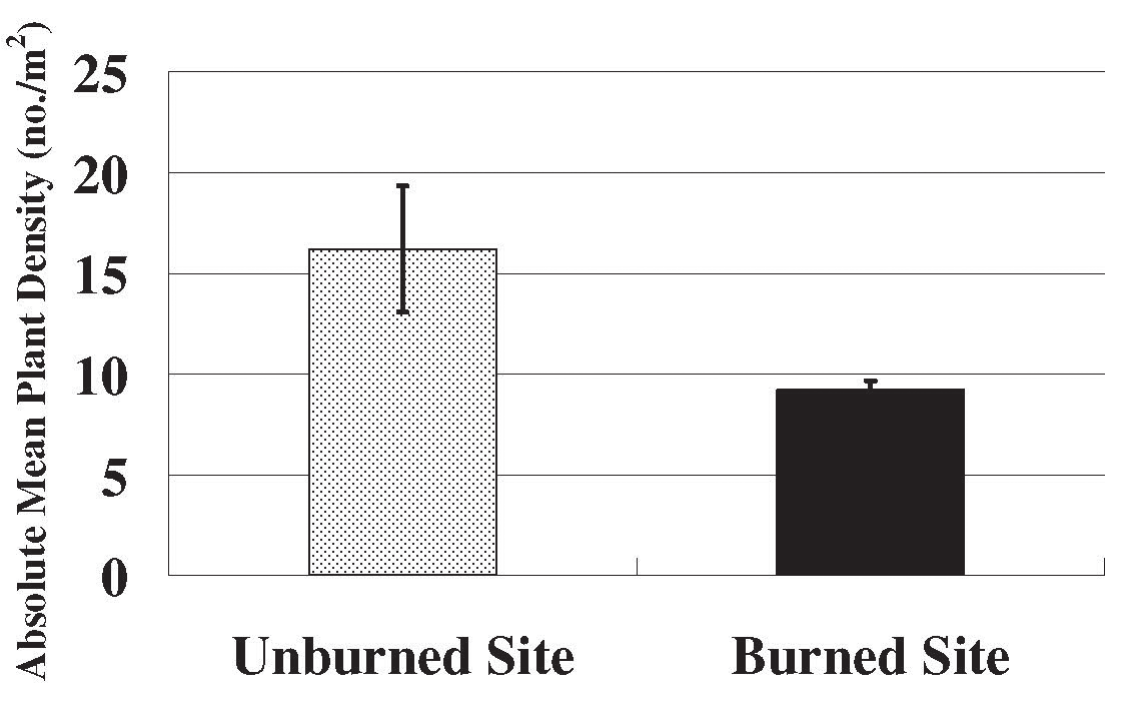
The plant density on the unburned site and the burned site per 1 m^2^. Error bars are ± SD (n=50). T-value = 6.75. P-value < 0.001.

**Figure 3.**
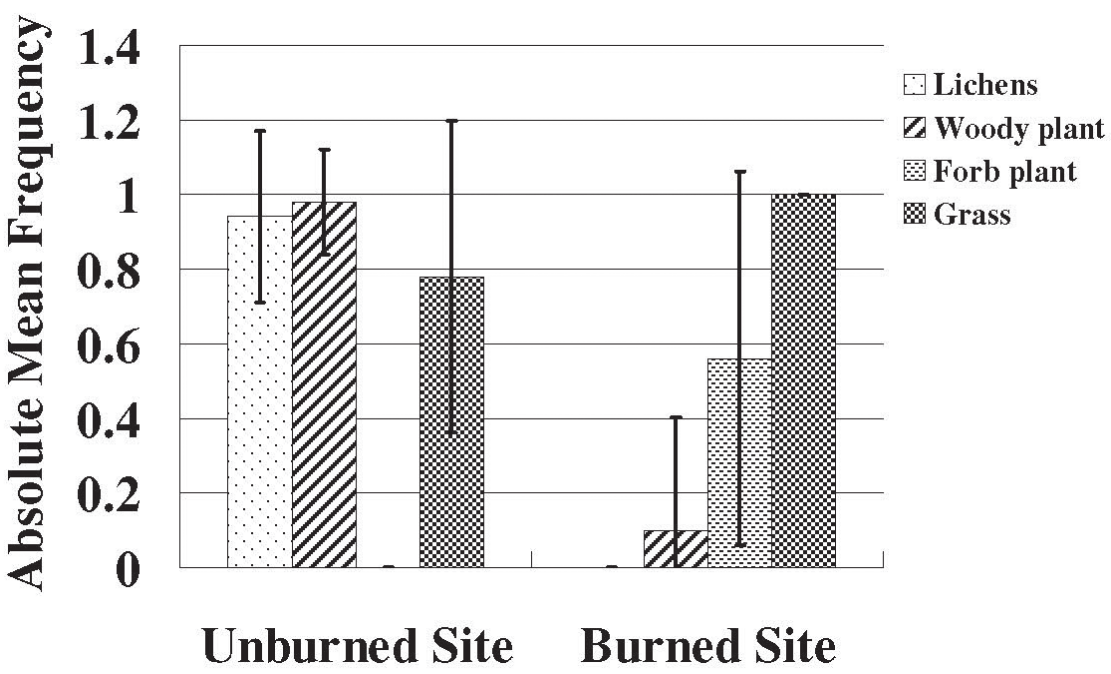
The frequency of lichens, woody plants, forb plants, grass on the unburned site and the burned site per 1 m^2^. Error bars are ± SD (n=50). Lichens: T-value = 27.71; P-value < 0.001. Woody plants: T-value = 18.96; P-value < 0.001. Forb plants: T-value = -7.90; P-value < 0.001. Grass: T-value = -3.72; P-value =0.001.

**Figure 4.**
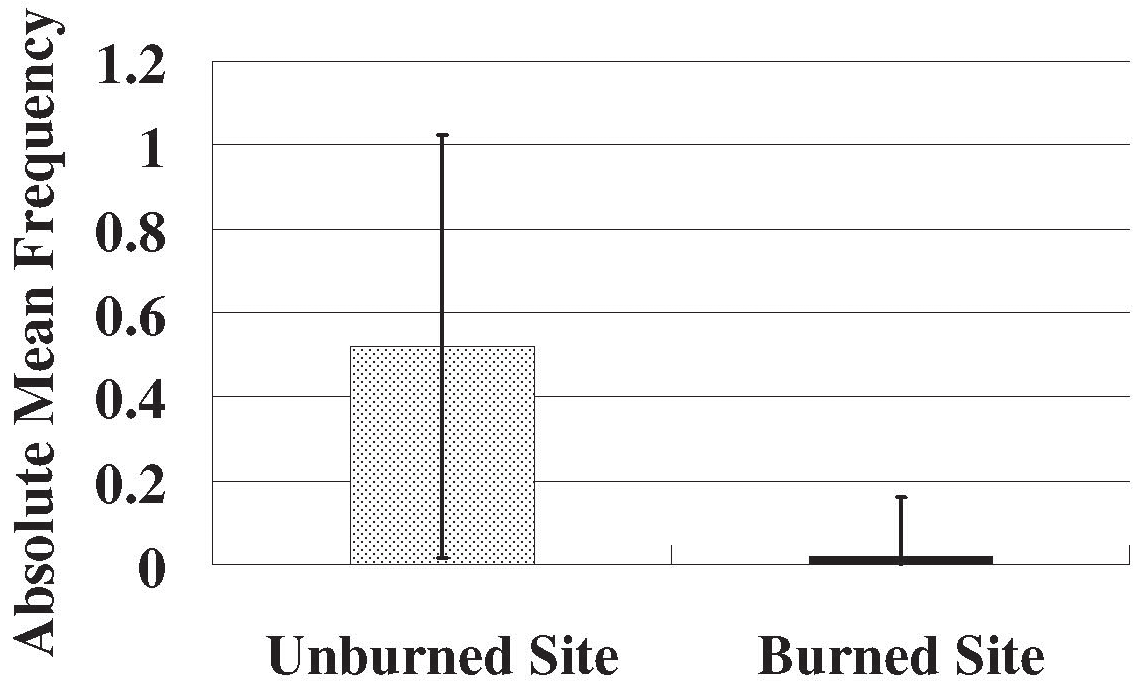
The frequency of native plants on the unburned site and the burned site per 1 m^2^. Error bars are ± SD (n=50). T-value = 6.50. P-value < 0.001.

**Figure 5.**
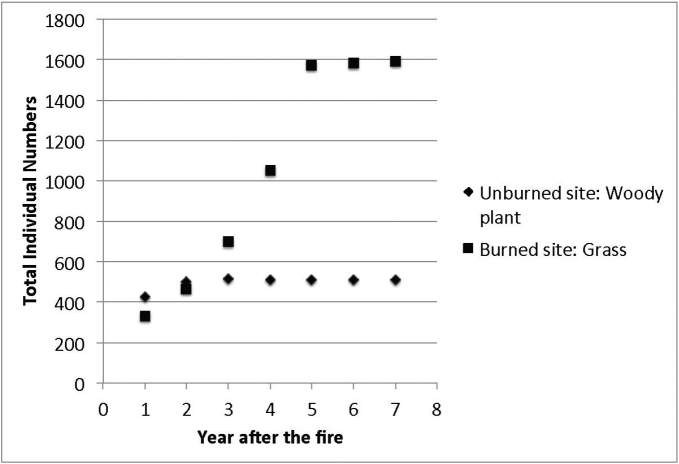
The total individual numbers of dominant species on the unburned site and the burned site from 2008–2015.

**Figure 6.**
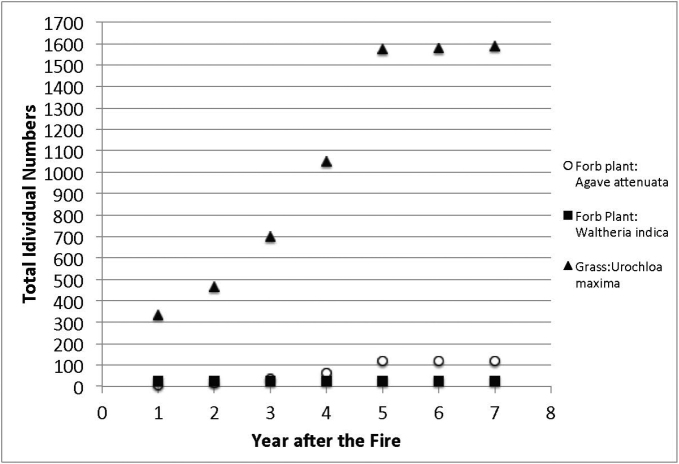
The total individual numbers of the top three dominant species on the burned site from 2008–2015.

**Figure 7.**
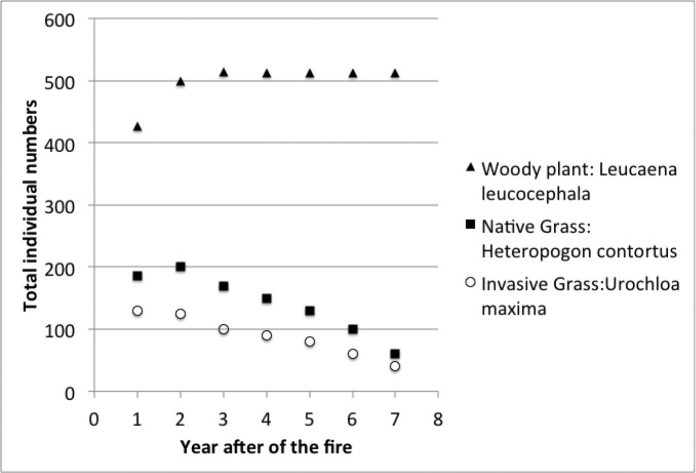
The total individual numbers of the top three dominant species on the unburned site from 2008–2015.

Species richness and plant density are greater on unburned sites than burned sites (Figs 1, 2) which supported the hypothesis. Frequency of lichens, woody plants, and native plants was greater on unburned sites than burned sites (Figs 3, 4). Frequencies of forbs and grasses are lower on unburned sites than burned sites (Fig. 3). All differences were significant between burned and unburned sites. Figure 5 shows the individual numbers of dominant species at the burned and unburned sites from 2008 to 2015. With increasing time, the gap between woody plants and grasses was getting larger. It appears the grass in burned site fills its niche 5 years after the fire but tree species in unburned site had not filled their niche yet. Figure 6 shows the major components in burned site. It indicates that among the three most abundant plants in burned site, *Agave* and *Waltheria* forbs were less abundant than the invasive grass. Figure 7 shows that in unburned plots, the tree frequency is similar from 2008 to 2015 with a positive trend. Native grass decreased but was not replaced by invasive grass, indicating woody plant encroachment in former native grass areas. In addition, if the species establish in the open canopy one year after the fire, not only are native species completely replaced by alien or invasive species, but competition for space becomes a limiting factor, particularly when the invasive grass achieves its optimal establishment after five years.

## Discussion

Understanding long-term succession and fire ecology is essential interpreting ecosystem fire responses and planning vegetation restoration. The results indeed supported the three hypotheses. The vegetation structure indicates that invasive plants were favored by the fire disturbance on Wa’ahila Ridge which created open spaces for pioneer species to establish seedlings more easily. Additionally, the environment surrounding the burned area was already dominated by invasive species which dominate the seed bank and few native species existed in the vicinity to contribute to the seed bank. Alien plants invasions in native ecosystems have become a topic of great concern in recent years, particularly in isolated island ecosystems such as the Hawaiian Islands (Loop and Mueller-Dombois 1989, Vitousek et al. 1987, McDaniel et al. 2008, Loope et al. 2013, Vorsino et al. 2014). More than 800 introduced plant species that have become naturalized in the Hawaiian Islands (Vitousek et al. 1987, Wagner et al. 1990, 2012). This study site is an example where invasion is a serious issue.

The invasive grass *Urochloa maxima* was the dominant species on burned sites, whereas the alien woody plant *Leuceana leucocephala* was the dominant species on unburned sites. However, a high proportion of the native grass *Heteropogon contortus*, and *Urochloa maxima* was also present on unburned sites. This indicates that after the fire *Urochloa maxima* has a better competitive ability than the woody alien species *Leuceana leucocephala* and the other alien grass species *Agave attenuata* to colonize quickly burned sites. Notably, many species occurred on burned sites that were not present on unburned sites. That suggests that the invasive plants on Wa’ahila Ridge have greater opportunity and ability to replace native plants in the short term after a fire.

*Leucaena leucocephala* is dominant in, and has highest frequency on, unburned sites. It indicates that this species has the ability to establish a large population on Wa’ahila Ridge. In addition, the native grass, *Heteropogon contortus* coexists equally with the invasive grass, *Urochloa maxima* on unburned sites, which infers that *Leuceana leucocephala* may play a critical role in the coexistence. In contrast, on burned sites, the invasive grass, *Urochloa maxima* has the highest frequency and other species have low frequency, which shows that the burned area is primarily occupied by the single species of *Urochloa maxima*. Year by year, the burned site became dominated this single invasive species, with scattered *Agave attenuata*, another dryland invasive species recently occurring in Hawai’i.

Although total species numbers on burned sites are higher than on unburned sites, the species numbers per plot are higher on unburned sites than burned sites. The results indicates that the species tends to be concentrated more in plots on the burned sites, but are more equally distributed over all plots on unburned sites. A possible explanation is that burned sites are dominated by grass and unburned sites are dominated by trees.

Plant density is greater on unburned sites. This indicates that a one-year recovery time is not enough for plant species to reach their maximum population density and burned materials may not provide enough nutrients to seedlings of the other types of plants, or after the fire disturbance invasive plants quickly occupy those open canopy and do not allow other species to dominate those areas. However, the plant density is trending towards similarity on burned and unburned sites year by year.

The results of this study support the findings of previous research on vegetation in the lowlands, which have a similar dynamic structure. For example, herbaceous species dominate the immediate post-burn environment, but most generally disappear after three to four years because they were shaded out by the recovering shrubs and trees in California (Keeley and Keeley 1981; Keeley et al. 1981). It has been documented that recurrent fires promote the presence of herbaceous species in Mediterranean type ecosystems in Spain and in California (Zedler et al. 1983, Faraco et al. 1993). Lichens have been promoted as a useful environmental indicator (Giordani et al. 2012, Li et al. 2013). No lichens exist on burned sites, but almost 100% of plots in the unburned sites have lichens. Records show that the unburned sites have not had any major disturbances for few years so that the pre-fire vegetative structure on Wa’ahila Ridge was well established (Armstrong 2015, Merinero et al. 2015). In the post-fire recovery phase, the first to third year after the fire played important roles in determining the probability of invasive or native tree, shrub, and grass recovery, contraction, or loss. (Ruiz et al. 2013, Pausas 2015). Dispersal affects the short-term and long-term persistence of patchily distributed species with patchily distributed resources in a highly temporally variable environment. Dispersal over even small distances reduce the opportunity of consequent density dependent interactions by moving seeds away from the immediate vicinity of parent plants but retaining them in a favorable area (Howe and Smallwood 1982, Schupp 1995). Longer-distance dispersal involves risk, as few seeds typically reach sites suitable for germination and growth (Venable and Lawlor 1980) although the most invasive species tend to be very good at long distance dispersal. However, according to other research reports, when local populations are subject to extinction or struggling to survive, in line with metapopulation theory, long-distance dispersal is required for populations to expand into new areas to discover better environments, and between-patch movement allows the persistence of subdivided populations (Hanski and Gilpin 1991, Cain et al. 2000, Howe and Miriti 2004). In this study, threatened native or endemic species were not observed establishing in the open canopy, indicating the competitive advantage of invasive or alien species.

In conclusion, this study provides primary data but fundamental information for policy makers develop appropriate conservation strategies to mitigate the serious loss of native habitats on O’ahu, Hawai’i and also provides useful information for island ecosystems and tropical vegetation ecology. Seven years of field data show the vegetation changes over time. It suggests the vegetation will not be recovering to the pre-fire state and even worse. Other studies have shown that post-fire vegetative structure does tend to return to its pre-fire state (Keeley 1986, Ireland and Petropoulos 2015), however, those are because of the environment is full of relatively high proportion native species. Thus, the content of native vs. invasive species around the fire disturbance plays a very significant role for vegetation succession. In this study, the pre-fire environment was already depauperate in native species and thus after the fire disturbance, the seed bank provided more invasive species than native species. Future studies should address spatial factors in studies of post-fire vegetation dynamics, as the spatial arrangement of patches in the landscape and the seed dispersal mechanisms are crucial processes influencing plant dispersal and colonization.
